# Transcatheter Aortic Valve Replacement in Elderly Patients: Opportunities and Challenges

**DOI:** 10.3390/jcdd10070279

**Published:** 2023-06-29

**Authors:** Bing Huang, Hui Yan, Yunyao Li, Qiping Zhou, Ayipali Abudoureyimu, Guiqiu Cao, Hong Jiang

**Affiliations:** 1 Department of Cardiology, Renmin Hospital of Wuhan University, Wuhan 430060, China; binghuang@whu.edu.cn (B.H.); 13193355319@163.com (Y.L.); 2 Cardiovascular Research Institute, Wuhan University, Wuhan 430060, China; yanhui1231666@163.com; 3 Hubei Key Laboratory of Cardiology, Wuhan 430060, China; 4 Department of Cardiology, Fifth Affiliated Hospital of Xinjiang Medical University, Urumqi 830000, China; zhouqiping0526@163.com (Q.Z.); ayipali@163.com (A.A.)

**Keywords:** transcatheter aortic valve replacement, transcatheter aortic valve implantation, aortic stenosis, elderly

## Abstract

Over the past two decades, the rapid evolution of transcatheter aortic valve replacement (TAVR) has revolutionized the management of severe aortic stenosis (AS) in the elderly. The prevalence of comorbidities in elderly AS patients presents a considerable challenge to the effectiveness and prognosis of patients after TAVR. In this article, we aim to summarize some of the clinical aspects of the current use of TAVR in elderly patients and attempt to highlight the challenges and issues that need further consideration.

## 1. Introduction

Aortic stenosis (AS) is a common valvular disease in the elderly population, comprising those aged 65 years or older, that exhibits a marked increase in prevalence with increasing age and is associated with substantially elevated mortality [1,2]. The overall frequency of AS in elderly adults is between 12% and 13%, while the prevalence of severe AS is approximately 2% to 4% [3,4]. Aortic valve replacement is the most effective treatment to alleviate the symptoms of stenosis and to maintain left ventricular function, although the risks associated with the procedure tend to increase with age. Of the elderly patients with severe AS, 40.5% of these patients were without surgical aortic valve replacement (SAVR) due to the high surgical risk, the patient’s preference, advanced age, and comorbidities [4,5]. Accordingly, transcatheter aortic valve replacement (TAVR) is a viable treatment option for the management of these elderly patients. With advances in TAVR technology, a minimally invasive, effective treatment for aortic valve disease is now available for many elderly and high-risk patients. In several important randomized controlled trials, TAVR was not inferior or even superior to SAVR in terms of mortality and improvement in cardiac symptoms in elderly patients with severe AS [6,7]. In the 2021 ESC guidelines on the management of severe AS, TAVR is recommended in older patients (≥75 years) or in those who are considered high-risk or unsuitable for surgery (class IA) [8].

**Figure 1:**
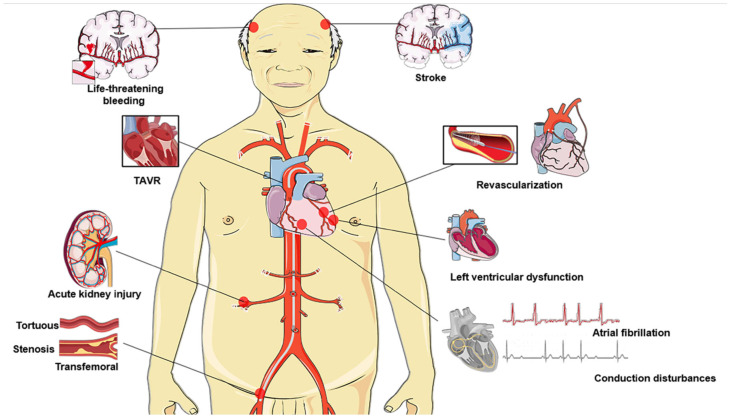
Impending issues and challenges among elderly individuals undergoing TAVR: ineligible for transfemoral access, timing of revascularization, atrial fibrillation, stroke, conduction disturbances prevention (indications for permanent pacemaker implantation), left ventricular dysfunction, acute kidney injury, life-threatening or major bleeding. TAVR, transcatheter aortic valve replacement.

The outcomes and safety of TAVR can be greatly improved by adequate preprocedural planning (i.e., regarding valve and access site selection) and imaging analysis (e.g., by multidetector computed tomography (MDCT), magnetic resonance imaging (MRI) and echocardiography), better patient selection, minimization of surgical procedures, and sufficient TAVR team experience. However, growing older is an important risk factor for numerous diseases, and elderly AS patients frequently have additional concurrent comorbidities. This presents a considerable challenge to the efficacy of TAVR and patient prognosis, which needs to be adequately understood and addressed to prolong and improve quality of life (Figure 1, Table S1). In this review, we aim to summarize some of the clinical aspects of the current use of TAVR in elderly patients and attempt to highlight the challenges and issues that need further consideration.

## 2. Access Site

The transfemoral (TF) approach is the most frequently applied route in TAVR and is indicated in more than 95% of elderly patients [6]. Preoperative MDCT has revealed that a small lumen diameter, dense and circumferential calcification, and severe tortuosity are common in this population, increasing the risk of access site complications [9]. As a result, alternative approaches to gain access must be evaluated, and the advantages and challenges of the currently used access routes are summarized in Table S2.

### 2.1. Trans-Subclavian/Axillary Arterial Access

SCA-TAVR is regarded as a favorable and less invasive alternative approach since it involves a short distance between the puncture site and the aortic annulus and is generally free of atherosclerotic calcification and significant tortuosity [10]. In the CoreValve trial, outcomes at 30 days and 1 year after treatment with SCA-TAVR were comparable to those in the TF group [11]. Similar to TF-TAVR, SCA-TAVR is restricted by vascular stenosis (<6 mm), substantial calcification and extensive tortuosity. In addition, a valve angle of >70 is considered a relative contraindication to SCA-TAVR [10,11].

### 2.2. Transaortic Access

Transaortic access is considered advantageous compared to the TF-TAVR and SCA-TAVR because it avoids the descending aorta and peripheral arteries in patients with extensive vascular disease, stenosis and excessive tortuosity. In addition, the access for placing the valve is independent of sheath size, and both CoreValve and SAPIEN valves are available for this approach. The multicenter prospective ROUTE study demonstrated the efficacy and safety of transaortic access implantation of SAPIEN XT and SAPIEN 3 transcatheter heart valves in elderly patients with multiple comorbidities [12]. However, transaortic access requires general anesthesia and surgical manipulation of the chest wall, thus limiting older patients with pre-existing pulmonary disease (such as pleural plaques or pleural disease) and pre-existing vascular conditions (such as aortic dissection or aneurysm at a prior aortotomy site) [10].

### 2.3. Transcarotid Access

For patients with a hostile chest and no other practical option, the transcarotid approach offers peripheral access without requiring the opening of the chest chamber. Chekrallah et al. showed that compared with the apical or aortic approach, transcarotid access is associated with the incidence of bleeding, atrial fibrillation (AF), and acute kidney injury (AKI) and can result in reduced the incidence of complications in elderly patients [13]. However, transcarotid access is not feasible in elderly people with carotid artery stenosis/occlusion, embolism, possible high-risk plaque or other anatomical challenges [14].

### 2.4. Transapical Access

Transapical access is the most invasive of the alternative routes to TAVR and is typically used in situations where the above approaches are contraindicated. The transapical approach requires direct access to the left ventricular myocardium, and the shorter path facilitates a better implantation angle, providing more control and precision for the operator. Because of the high surgical invasiveness, transapical access is associated with a marked risk of complications [15]. Two propensity-score-matched studies showed that older patients treated with transapical access had higher rates of short- and long-term mortality and major bleeding, longer hospital stays, and slower recovery, despite similar stroke rates at 30 days after surgery in both groups [16,17]. Furthermore, the frequent presence of prior myocardial infarction or calcification, severe pulmonary disease, and poor cardiac function in elderly AS patients makes the transapical access infeasible [10].

Although the femoral approach remains the preferred option for most patients, alternative access locations have been shown to be safe for elderly patients who are not candidates for TF-TAVR. The selection of the optimal approach requires knowledge of the characteristics of the approach and adequate preoperative assessment of the patient’s aortic and vascular anatomy.

## 3. Coronary Artery Disease

Coronary artery disease (CAD) is a common comorbidity in patients with severe AS, and both conditions have similar risk factors, with CAD affecting more than 50% of AS patients over 70 years of age [18,19]. The mortality rate in CAD patients within 30 days of TAVR was 10.1 times higher than that in non-CAD patients (95% CI: 2.1–174.8) [20]. Stefanini et al. used the SYNTAX score to assess the severity of CAD in elderly patients with severe AS undergoing TAVR. They discovered that patients with a SYNTAX score >22 had a greater risk of cardiovascular death, stroke, or myocardial infarction after one year of TAVR, and the severity of CAD was associated with a high risk and poor clinical outcome at baseline [21]. Similar findings were reported in an Israeli multicenter clinical trial [22].

### The Timing of Revascularization

The link between severe CAD and mortality may be decreased by more complete revascularization prior to TAVR. Percutaneous coronary intervention (PCI) is thought to be safer and more practicable for elderly TAVR patients at high risk for reperfusion surgery, and up to 25% of TAVR patients receive PCI during preoperative exams or TAVR surgery [23]. The prospective SURTAVI trial involved comparing the primary endpoint (the rate of all-cause mortality and disabling stroke at 2 years) in elderly patients with severe AS and noncomplex CAD (SYNTAX score ≤ 22) treated with TAVR + PCI and SAVR + coronary artery bypass grafting (CABG). After the randomization of treatment, no significant difference in the primary endpoint was found between the two groups [24]. In a subsequent meta-analysis, the 30-day safety outcome and 2-year mortality did not significantly differ between the two options [25]. Unfortunately, several of the studies cited by the authors differed in terms of revascularization strategies, accuracy of surgical risk assessment, and classification of CAD using the SYNTAX score.

However, the therapy of SAVR + CABG or TAVR + PCI and the timing of PCI are not specifically advised in the guidelines for elderly TAVR and revascularization. Both Patterson T and Altibi AM et al. showed that patients who underwent PCI before TAVR were not associated with rehospitalization or improved 1-year mortality [26,27]. Although it was found in a single-center study that concomitant TAVR + PCI reduced the SYNTAX score (from 8.0 ± 5.7 to 3.0 ± 4.9) in patients with severe AS and highly significant coronary artery disease, there was a significant increase in overall mortality at 3 years after TAVR [28]. The high surgical risk and comorbidity characteristics of this group of patients may explain this outcome. On the other hand, there was a greater incidence of in-hospital mortality and complications in patients who underwent PCI and TAVR during the same hospital stay [29]. Then, a meta-analysis demonstrated that there was no additional clinical advantage in terms of important clinical outcomes for elderly patients with pre/combined PCI + TAVR for severe AS and CAD [30]. In the RE-ACCESS (Reobtain Coronary Ostia Cannulation Beyond Transcatheter Aortic Valve Stent) study, 7.7% of patients experienced unsuccessful coronary cannulation after TAVR. The combination of the Evolut transcatheter aortic valve, a higher transcatheter aortic valve–sinus of Valsalva relation, and implantation depth were independent factors for coronary cannulation failure after TAVR [31]. 

Additionally, bleeding and vascular damage problems after TAVR were considerably more common in patients who had PCI within 30 days of the procedure [32]. Revascularization with PCI prior to TAVR was associated with an increased likelihood of bleeding 30 days after TAVR [26,27]. Although avoiding multiple procedures and lowering the risk of obtaining vascular access at various times are advantages of simultaneous combined procedures, additional vascular puncture, contrast injection, and antiplatelet therapy for staged PCI before TAVR may raise the likelihood of problems after TAVR in senior individuals [23], especially in patients with complex CAD.

The history of CAD and timing of revascularization are important challenges in elderly patients with TAVR. Thus, additional randomized studies are required to more clearly define the clinical practice for myocardial revascularization in older patients undergoing TAVR for AS.

## 4. Atrial Fibrillation

The most prevalent persistent arrhythmia in elderly individuals is AF, which affects 10% or more of the global population ≥80 years [33]. The prevalence rates of pre-AF and new-onset AF (NOAF) in elderly patients with AS after TAVR were approximately 14.4–40.7% and 7.2–16.1%, respectively [34,35,36]. NOAF was linked to a higher risk of 30-day mortality, AKI, bleeding, stroke, and heart failure hospitalization [34,37]. Concomitant diseases in the elderly and frail populations may explain the high prevalence of these events. 

### 4.1. Anticoagulation

Anticoagulants are recommended to reduce the risk of stroke in TAVR patients with AF despite the risk of bleeding [8,38]. Participants who started anticoagulant therapy immediately after receiving a diagnosis of NOAF had a significantly reduced 30-day risk of stroke and systemic embolism than patients who waited longer [38]. The advantages of non-vitamin K oral anticoagulants (NOACs) over vitamin K antagonists (VKAs) in elderly patients with AF have been widely documented [39,40]. In contrast, in the elderly TAVR population with AF, current observational studies and randomized controlled trials have shown conflicting results. In the France-TAVI and FRANCE-2 registries, among the 8960 patients treated with OACs, 2180 (24.3%) received NOACs (apixaban (52.5%), rivaroxaban (35.4%), and dabigatran (12.1%)). After a period of follow-up, the long-term mortality (HR: 1.37; CI: 1.12–1.67; *p* < 0.005) and major bleeding (HR: 1.64; 95% CI: 1.17–2.29; *p* < 0.005) were lower in elderly patients on NOACs [41]. On the other hand, the primary result and primary safety endpoint in the ATLANTIS randomized controlled trial did not differ between patients who received NOACs (apixaban) and VKA [42]. The ENVISAGE-TAVI AF trial yielded comparable results, and NOACs (edoxaban) were not inferior to VAKs in the composite main outcome of severe clinical events; however, the rate of important gastrointestinal bleeding in edoxaban was higher [43]. Finally, a recent meta-analysis also showed that the use of NOAC therapy was similar to VKA treatment in reducing all-cause mortality, stroke, and severe and/or life-threatening bleeding episodes in older patients who required OACs after TAVR [44]. Furthermore, age and comorbidities are predictors of mortality, stroke, and bleeding events over time and should be taken into account when deciding on the best antithrombotic therapy [38].

### 4.2. Left Atrial Appendage Occlusion

The risk of ischemic stroke or systemic embolism was reduced with concurrent left atrial appendage occlusion (LAAO) performed during the procedure compared to that without LAAO [45]. In a recent case study, simultaneous TAVR and LAAO were found to be a realistic and secure combination for patients with severe AS and AF who were deemed ineligible for OACs [46]. LAAO after TAVR may be an option for long-term oral anticoagulation medication in elderly patients with contraindications to anticoagulation, high bleeding risk, high risk of drug interactions, or the need for dual antiplatelet therapy due to coronary stent implantation. In addition, the careful monitoring of the patient’s electrolyte and volume changes, pain management, and anemia treatment may all help to some degree to prevent AF [47].

In summary, the use of anticoagulation strategies and LAAO for elderly patients with TAVR combined with AF is reasonable, but more data are urgently needed to optimize antithrombotic regimens in TAVR patients with AF.

## 5. Stroke

### 5.1. Incidence and Prognosis of Stroke

One of the harshest and most harmful adverse effects of TAVR is stroke, which has a direct correlation with severe disability and high death. The incidence of stroke or transient ischemic attack in individuals 30 days and 1 year after TAVR was found to be 0.6–6.7% and 2.2–10.4% in pivotal randomized TAVR trials [48,49,50]. Most early strokes occurred in the first 3 days after TAVR. The mortality in elderly patients with stroke after TAVR (within 30 days) was approximately 6.1 times higher than in patients without stroke [51]. Early stroke could be caused by intraoperative mechanical injury (such as excessive expansion, atherosclerotic or calcified fragments, tissue fragments of the aorta or left ventricle), inadequate anticoagulation management, NOAF, acute renal injury, chronic kidney disease, history of stroke and falls, and important vascular complications [52,53]. Within a year after TAVR, thrombosis and embolism may develop due to various biological reactions to the aortic prosthesis and its materials, history of stroke, chronic AF, atherosclerotic risk, and patient frailty [35,53,54]. In addition, TAVR is associated with a high incidence of clinically asymptomatic cerebral embolism, as revealed by diffusion-weighted MRI of the brain [55]. These subclinical phenomena may be associated with progressive cognitive deterioration, leading to neurocognitive decline and dementia [56].

### 5.2. Preventive Measures for Stroke

Protective devices to avoid embolism, antithrombotic therapy, and the adjustment of surgical factors associated with TAVR should all be used as preventive methods for stroke after TAVR. Cerebral embolic protection devices (CEPDs) reduce the quantity of embolic materials that enter the cerebral arteries during surgery, hence reducing the risk of stroke and the severity of nerve injury [57,58]. Depending on their mode of operation, CEPDs are classified into two groups: devices that encourage fragments away from the branch of the aortic arch and devices that trap fragments before they reach the cerebral artery (in whole or in part) [57,58]. The SENTINEL TCEP device study evaluated the safety and effectiveness of the sentinel cerebral protection system in elderly TAVR patients [59]. However, the incidence of all-cause stroke and the median of the total volume of new lesions in the protected area within 30 days were not significantly reduced (*p* > 0.05), even though the author discovered granular fragments in the filter and confirmed the safety of device implantation in the majority of patients [59]. Similarly, both the REFLECT I (TriGuardTM HDH) and REFLECT II (TriGUARD 3) clinical trials met their primary safety endpoints but did not meet the efficacy endpoints including death or 30-day stroke rate [58,60]. The PROTEMBO C Trial revealed that compared to the predetermined performance target, the incidence of major adverse cardiocerebrovascular events was lower and the technical success rate was higher in patients who had the ProtEmbo brain protection system 30 days after TAVR; in comparison to earlier series of studies, the volume of brain lesions on diffusion-weighted MRI was smaller [61].

The safety of CEPDs has been demonstrated, but there is a lack of clear evidence supporting their efficacy in stroke prevention. Therefore, updated CEPDs and additional randomized controlled trials are still required in the future to validate the therapeutic effects of CEPDs in elderly TAVR patients.

## 6. Conduction Disturbances

### 6.1. Incidence

The high prevalence of baseline conduction abnormalities in elderly individuals with many comorbidities may help to partially explain the high incidence of conduction disturbances after TAVR. A high degree atrioventricular block (HAVB) and new-onset left bundle branch block (LBBB) are the most frequent conduction abnormalities after TAVR in elderly individuals. The incidence of new-onset LBBB continues to change as transcatheter heart valve devices and operating practices improve, with the rate ranging from 6% to 77% in the newer generation of devices [62,63]. The prevalence of HAVB at 30 days and 1 year in patients after TAVR was approximately 5–10% and 10–15% [64,65,66]. Primarily as a result of the different implantation depths and expansion mechanisms of the two valves, the self-expanding Evolute R and PRO exhibited higher LBBB and pacemaker implantation rates (PPI) than the balloon-expanded devices SAPIEN 3 and SAPIEN 3 ULTRA [67,68,69]. Most cases of new-onset LBBB appeared in the perioperative period, with approximately half of these patients experiencing resolution before discharge and a few patients presenting with persistent LBBB at 30 days and 1 year later [62,70,71].

### 6.2. Prognosis

In two meta-analyses, it was reported that the rate of PPI associated with new-onset LBBB was approximately two-fold higher during the interim follow-up (1–2 years) [72,73]. The substantial impact of new-onset LBBB on the increased risk of HAVB and PPI has been consistently reported, and in the majority of studies, HAVB is reported to be the primary indicator of PPI rates at follow-up [70]. The differences in PPI rates and impact observed in patients with new-onset LBBB after TAVR may be due to a lack of consensus and different clinical management strategies across centers. In addition, the long-term effects and results of new-onset LBBB have been widely debated, and different studies have produced conflicting findings regarding cardiovascular and overall mortality. Nevertheless, both the PARTNER II trial and meta-analyses have demonstrated that new-onset LBBB in the elderly was associated with increased all-cause mortality, cardiovascular mortality, rehospitalization and decreased left ventricular systolic function in the 1–2 years of follow-up after TAVR [70,73].

### 6.3. Risk Factors

Preoperative assessment of the aortic root anatomy by MDCT in elderly patients may help identify patients at risk for new onset LBBB. A shorter membranous septal length, left ventricular outflow tract eccentricity, annular oversizing, and deeper implant depth are considered independent predictors of the occurrence of new-onset LBBB after TAVR [74]. In addition, other clinical and ECG predictors include the female sex, diabetes, prior coronary artery bypass grafting, preoperative conduction abnormalities, aortic valve calcification, and prolonged QRS (>150–160 ms) and PR intervals (>240 ms) [74,75].

The current data do not support the indication of systemic prophylactic PPI (in the absence of significant bradyarrhythmia) in patients with new-onset LBBB after TAVR. Several studies have used ambulatory electrocardiography to monitor risk factors for conduction block in discharged patients and to guide the safety of PPI [64,65,66]. On the other hand, several studies have proposed the use of invasive electrophysiological studies (EPSs) to stratify the risk of HAVB and to guide the implantation of PPIs after TAVR. Although studies with this strategy typically have limited sample sizes, the results of these studies show good negative predictive values for EPS after TAVR, but slightly lower positive predictive values [76,77]. EPSs represent a potentially valuable tool to evaluate these patients, and further large-scale studies are needed to further validate this approach.

Transcatheter heart valve system deployment in elderly patients often damages the specialized electrical system of the heart, resulting in new conduction disturbances that have deleterious effects on cardiac function and patient prognosis. Conduction block following TAVR may be reduced to some extent by an adequate preoperative risk evaluation, the modification of procedural factors (e.g., higher implant depth and less annular oversizing), and close monitoring of ECG changes.

## 7. Low-Flow, Low-Gradient Aortic Stenosis

The results of retrospective studies have shown that the prevalence of symptomatic left ventricular dysfunction (LVEF < 50%) in elderly patients with severe AS is 24–35% [78,79]. A lower LVEF may cause a “low-flow (LF), low-gradient (LG)” phenomenon that is significantly associated with an increased risk of surgical death. LF is an independent predictor of mortality in patients with severe AS (HR ≈ 1.5) [80]. Patients with LF–LG have the highest risk of mortality and adverse events among all patients with severe and symptomatic AS, with a 2-year survival rate of approximately 40–60% with medical management [81,82,83]. The recent PARTNER trial involved analyzing the prognosis of 984 elderly patients with LF AS based on the stroke volume index, and patients with severe had the lowest mean transvalvular pressure and higher 1-year all-cause mortality (26.5%) [84].

### Prognosis and Risk Factors

Previous studies have revealed that aortic valve replacement improves mortality and clinical functional status in patients with LF–LG, even in AS patients with a low LVEF. However, the mortality in these patients has remained high, with a perioperative mortality rate of 16–22%, a 1-year mortality rate of approximately 20%, and a long-term mortality rate of 22–50% [80,83,85,86]. In patients with LF–LG AS, TAVR significantly improved 2-year mortality compared with pharmacological treatment (45.9% vs. 76.2%, *p* < 0.001). Among high-risk patients, the post-6-month mortality rate was also significantly lower in the TAVR group than in the SAVR group (15.6% vs. 24.7%; *p* = 0.04) [80]. In the current literature, it is reported that the prognosis of patients with LF–LG AS after TAVR was significantly worse than that of patients with a high gradient and paradoxical LF–LG AS [80,83]. Eleid MF et al. showed that a reduced stroke volume index (<35 mL/m^2^), reduced LVEF (<50%), and low gradient (<40 mm Hg) were associated with increased mortality at 1 year after TAVR [87]. Consistent with most reports, there was a relatively high proportion of LF–LG patients (16.9%) in the study by Castelo A et al. Patients with LG (LVEF < 40%) after TAVR had the worst prognosis and a higher overall mortality (*p* = 0.035) and cardiovascular mortality (*p* = 0.038) compared with patients with a high gradient [88]. This phenomenon may be attributed to the previous history of myocardial infarction, cardiac amyloidosis, diabetes mellitus and renal dysfunction in elderly high-risk patients; these conditions favor the development of left ventricular dysfunction in this group of patients [88,89].

A history of significant CAD, preoperative mean gradient, and dobutamine stress echocardiography results in reduced ejection fraction LF–LG patients were identified as independent predictors of perioperative mortality after TAVR [90]. A low-dose dobutamine-loaded echocardiogram is recommended to differentiate between true-severe and pseudo-severe aortic stenosis (increase in valve area to >1.0 cm^2^ with increased flow) and to assess left ventricular systolic reserve function [91]. However, the application to elderly patients has only been evaluated in small areas.

In summary, TAVR is a viable option in the treatment of elderly AS patients with LF–LG, whereas adequate preoperative evaluation still needs to be universal. Further research data and advanced technology or newer management approaches are needed to ensure that more patients benefit.

## 8. Acute Kidney Injury

AKI occurs in up to 3–57% of the elderly undergoing TAVR and is a powerful predictor of mortality, longer hospital stays, and higher costs [92,93,94]. In previous studies, it has been reported that TAVR is associated with a significantly lower frequency of AKI and hemodialysis compared to SAVR, even among patients without chronic kidney disease. The presence of AKI is associated with an increased risk of short- and long-term mortality, which correlates with its severity [92].

### 8.1. Risk Factors

Perioperative bleeding is a strong risk factor for AKI in patients treated with TAVR, and the negative effect on renal function could be partially explained by reduced renal perfusion [92]. In addition, the choice of different TAVR access sites and valve types also affects the occurrence of postoperative AKI. Indeed, compared with the transfemoral approach, the transapical approach was significantly associated with an increased risk of AKI after TAVR (OR 3.20; 95% CI 1.68–4.36) [95]. Similarly, Strauch et al. showed that up to 21% of patients with AKI after TAVR (transapical access) required hemodialysis [94]. Finally, in the SOLVE-TAVI trial, the number of fluoroscopy exposures and total contrast dose were higher in the self-expandable valve group than in the balloon-expandable valve group. There was no significant difference in the incidence of AKI between the two groups [96]. Independent predictors of AKI reported in the literature include pre-existing coronary or cerebrovascular disease, chronic kidney disease, respiratory failure, and transfusion [97,98]. However, the clinical application value of these predictors still needs to be verified in large areas.

### 8.2. Prevention of Acute Kidney Injury

At present, the main clinical prevention method is to improve the preoperative condition of patients and control the underlying diseases. Close hemodynamic monitoring during the perioperative period may also allow the early detection of hypovolemic and low-cardiac-output states to prevent the development of AKI. In addition, ultrasound as an alternative to angiography for guiding arterial access cannulation may further reduce the occurrence of AKI after TAVR. However, research on the prevention of AKI is still relatively scarce overall, and specific prevention methods need to be further explored.

## 9. Bleeding

A high incidence of life-threatening (15%) and major bleeding (20%) after TAVR has been reported in the earlier literature [99], and complications are expected to decrease substantially with the further development of TAVR techniques. In second-generation TAVR devices, life-threatening and major bleeding after TAVR occurred in approximately 3.9% and 8.3% of TAVR procedures, respectively [100]. Life-threatening or major bleeding was strongly associated with a higher 1-year mortality (HR: 2.09; 95% CI: 0.98–4.42) [101].

The occurrence of major bleeding complications after TAVR was associated with vascular complications and significant surgical complications. In the PARTNER I randomized trial, major vascular complications (HR:11.64, 95% CI: 6.83–19.87) and important surgical complications leading to the use of hemodynamic support (HR: 2.56, 95% CI: 1.18–5.56) were identified as the strongest independent predictors of major bleeding complications [102]. Moreover, high BMI values and the low strength of fibrin clots measured with thromboelastography immediately after TAVR were determined to be independent predictors of severe and life-threatening bleeding complications in elderly patients in the short term [103,104]. Another important factor affecting postoperative bleeding is improper anticoagulation therapy. Although many studies have confirmed the safety and effectiveness of OACs in elderly TAVR patients, the risk of postoperative bleeding may increase for high-risk patients with multiple complications of AS.

Vincent F et al. showed that ultrasound guidance effectively reduced vascular complications and bleeding complications for transfemoral TAVR [101]. At the same time, relevant studies on the prevention of postoperative bleeding have also shown that the application of protamine and percutaneous plugging devices can reduce the incidence of postoperative bleeding [105,106]. There is still a very broad research prospect for the related technologies to prevent postoperative bleeding, which may help to reduce postoperative bleeding in elderly patients and improve prognosis.

## 10. Vascular Complications

Vascular complications (VCs) frequently occur after TF-TAVR and have a significant impact on poor outcomes in elderly patients [107]. VCs are not only closely associated with prolonged hospitalization, a poorer quality of life, and in-hospital and 30-day mortality outcomes, but also with bleeding-related complications [107,108]. In the PARTNER trial (B and A), 15.3% of patients had major VCs and 11.9% had minor VCs within 30 days after TAVR [109]. These rates were lower in more recent clinical trials of intermediate-risk patients treated with second-generation, lower-profile delivery systems [110]. Similarly, the TVT registry data demonstrated a decline in overall VCs and in-hospital bleeding events over time after TAVR from 2011 to 2016 [107]. VCs and bleeding events are caused by several procedural and patient-related variables, such as the female sex, peripheral arterial disease, sheath size (>17 Fr), severe iliofemoral tortuosity, and open surgical cutdown [107,108]. In addition, the experience of the TAVR team and center as a whole plays an important role and affects the incidence of VCs [109].

An overview of common VCs and their clinical signs, risk factors, and management strategies is depicted in Table S3. At the same time, considerable emphasis has been placed on reducing the size of transcatheter valve systems, improving percutaneous closure and hemostasis of the primary access site [111,112]. Therefore, the main methods to avoid VCs are to enhance the preoperative evaluation, choose a reasonable method for cutdown or percutaneous preclosure, and evaluate alternative access routes if necessary.

## 11. Future Directions and Conclusions

Although the updated ESC guidelines promote TAVR as the primary treatment strategy for patients with AS aged 75 years and older, the management of elderly patients with AS with many comorbidities remains a major challenge. Further additional data from randomized studies are needed on currently controversial issues, including optimal vascular access, the timing of revascularization, better antithrombotic therapy, the efficacy of CEPDs, the mismanagement of LF–LG AS, and indications for PPI therapy. At the same time, the improvement of complications associated with TAVR (stroke, VCs, bleeding-related, AKI, AF, and conduction abnormalities) will require procedural and/or technical improvements. In addition, the individual design features of each device system remain a challenge for optimal transcatheter heart valve device selection. Unique patient characteristics and anatomically relevant factors often influence the choice of transcatheter heart valve system, which depends heavily on expert opinion and operational experience. Therefore, adequate preoperative imaging analysis to assess the anatomy of the patient’s valve and the complexity of the vascular access plays a crucial role in TAVR and the prevention of perioperative complications. In the future, with more imaging techniques and updates to TAVR devices, the indications for TAVR may be relaxed and the clinical outcomes further optimized.

## Data Availability

Not applicable.

## Supplementary Materials

The following supporting information can be downloaded at: https://www.mdpi.com/article/10.3390/jcdd10070279/s1, Table S1: Clinical outcomes at 30 days and 1 year for the elderly in the pivotal randomized TAVR trials; Table S2: Advantages and challenges of alternative access routes; Table S3: Vascular complications: clinical signs, risk factors and management strategies.

## Abbreviations

AKI = acute kidney injury; AS = aortic stenosis; AF = atrial fibrillation; CEPDs = cerebral embolic protection devices; CABG = coronary artery bypass grafting; CAD = coronary artery disease; HAVB = high degree atrioventricular block; LBBB = left bundle branch block; LVEF = left ventricular dysfunction; LF = low-flow; LG = low-gradient; MDCT = multidetector computed tomography; MRI = magnetic resonance imaging; NOAF = new onset atrial fibrillation; NOACs = non-vitamin K oral anticoagulants; PCI = percutaneous coronary intervention; PPI = permanent pacemaker implantation; SAVR = surgical aortic valve replacement; TAVR = transcatheter aortic valve replacement; TF = transfemoral; SCA = trans-subclavian/axillary arterial; VKAs = vitamin K antagonists.
