# Supplementary material for: Transcatheter Aortic Valve Replacement in Elderly Patients: Opportunities and Challenges
Source: J Cardiovasc Dev Dis. 2023 Jun 29;10(7):279. doi: 10.3390/jcdd10070279 (PMC10380827; doi:10.3390/jcdd10070279)
Supplement: Supplementary file 1 [file jcdd-10-00279-s001.zip › jcdd-2394192-supplementary.pdf]

Table S1 Clinical outcomes at 30 days and 1 year for the elderly in the pivotal randomized TAVR trials.

	PARTNER B	PARTNER A	U.S. CoreValve	PARTNER 2	SURTAVI	PARTNER 3	Evolut Low Risk
**Number**	NCT00530894	NCT00530894	NCT01240902	NCT01314313	NCT01586910	NCT02675114	NCT02701283
**Year**	2010	2011	2014	2016	2017	2019	2019
**Age (mean ± SD)**	83.6±6.8	83.1±8.6	83.2±7.1	81.5±6.7	79.9±6.2	73.3±5.8	74.0±5.9
**Sample size**	179	348	390	1011	864	496	734
**Death from any cause (%)**							
30 days	5	3.4	3.3	3.9	2.2	2-3	0.5
1 year	30.7	24.2	14.2	12.3	6.7	1	2.4
**Death from any cause or disabling stroke (%)**							
30 days	--	--	5.9	6.1	2.8	--	0.8
1 year	--	--	16.3	14.4	28.1	8.5	2.9
**Death from cardiac causes (%)**							
30 days	4.5	3.2	3.1	3.3	2	--	0.5
1 year	19.6	14.3	--	7.1	4.8	0.8	0.7
**Stroke or transient ischemic attack (%)**							
30 days	6.7	5.5	5.7	6.4	4.5	0.6	4
1 year	10.6	8.3	10.4	10.4	8.2	2.2	5.8
**Major vascular complication (%)**							
30 days	16.2	11	5.9	7.9	6	--	3.8
1 year	16.8	11.3	6.2	8.4	--	--	3.8
**Life-threatening or Major bleeding (%)**							
30 days	16.8	9.3	28.1	10.4	12.2	3.6	2.4
1 year	22.3	14.7	29.5	15.2	--	--	3.2
**Acute kidney injury Creatinine >3 mg/dl (265 μmol/liter) (%)**							
30 days	0	1.2	6	0.3	1.7	--	0.9
1 year	1.1	3.9	6	3.4	--	--	0.9
**Myocardial infarction**							

30 days	0	0	2.3	1.2	0.9	--	0.9
1 year	0.6	0.6	2.3	2.5	2	--	1.7
**Coronary artery obstruction (%)**							
30 days	--	--	--	0.4	0.2	--	0.9
1 year	--	--	--	0.4	--	--	0.9
**NOAF (%)**							
30 days	0.6	8.6	11.7	9.1	12.9	5	7.7
1 year	0.6	12.1	15.9	10.1	--	7.2	9.8
**New pacemaker (%)**							
30 days	3.4	3.8	19.8	8.5	25.9	5	17.4
1 year	4.5	5.7	22.3	9.9	--	--	19.4

NOAF: new-onset atrial fibrillation

Table S2 Advantages and challenges of alternative access routes

Access routes	Advantages	Challenges
**Transapical**	Fewer VCs<br>Less contrast and fluoroscopy<br>Short distance from sheath to annulus	Invasive<br>Myocardial injury<br>Higher rates of mortality and major bleeding<br>Longer hospital stays<br>Delayed bleeding<br>Pseudo-aneurysm formation<br>Ventricular injury
**Transaxillary/subclavian**	Favored alternative access<br>Less invasive<br>Free of prohibitive<br>Atherosclerotic calcification and significant tortuosity<br>Short distance from sheath to annulus	Increased risk of vessel injury<br>Challenging to control bleeding<br>Restricted by vessel caliber and calcification<br>Not advisable for patients with significant vascular tortuosity or calcification, or arterial diameter less than 6-7 mm
**Transaortic**	Direct visualization of the aorta<br>Avoidance of left ventricular apex<br>Fewer vessel injuries<br>Avoidance of tissue injury in patients with friable myocardium	Invasive, requires sternotomy or a right minithoracotomy for access<br>Use with caution in patients with prior sternotomy or bypass grafts that overlie aorta
**Transcarotid**	Percutaneous option<br>Less invasive<br>Straightforward procedure	Potential risk of stroke<br>Not indicated for elderly patients with carotid stenosis/occlusion, risk of embolism and high-risk plaque

VCs: Vascular complications.

Table S3 Vascular complications: clinical signs, risk factors and management strategies

	**Clinical signs**	**Risk factors**	**Management strategies**
**Aortic complications**			
Aortic rupture	Hemopericardium<br>Pericardial tamponade	Atheromatous and/or tortuous<br>Oversizing of the valvuloplasty balloon or prosthesis<br>Severe annular calcification	Immediate surgery<br>Aortic occlusion balloon<br>Endovascular repair using covered stent-grafts
Aortic dissection	Acute/subacute chest/abdominal pain<br>Neurological or hemodynamic changes	Damage to the aortic root and ascending aorta by balloon, valve or delivery system<br>Catheters or guidewires may damage the intima	Surgical and endovascular<br>Medical management
**Iliofemoral complications**			
Access site bleeding/Hematoma	Access site bleeding,<br>Persistent pain<br>Swelling	Female sex<br>Renal failure<br>Peripheral arterial disease<br>Sharp artery angulation	Majority resolve spontaneously or with manual compression<br>Anticoagulation reversal<br>Prolonged balloon angioplasty<br>If a larger injury occurs, stent graft should be implanted
Arterial dissection	Limb ischemia<br>Hemodynamic deterioration	Arterial calcification and/or tortuous	Prolonged balloon angioplasty<br>Sent implantation or surgical treatment
Arterial perforation	Back pain (retroperitoneal hematoma)<br>Leg hematoma	Arterial calcification<br>High-profile sheath systems and sheath delivery systems	Immediate reversal of anticoagulation<br>Prolonged balloon angioplasty<br>Open surgical repair and endovascular stenting
Pseudoaneurysms	Painful<br>Pulsatile groin mass	Advanced age, frailty, high BMI<br>Current anticoagulation medication<br>Use of high-profile sheaths<br>High/low puncture<br>Arterial and venous puncture<br>Severe vascular calcification<br>Failed manual compression	Observation if ≤3-3.5 cm<br>Ultrasound-guided compression and thrombin injection, interventional or surgical occlusion if ≥3.5 cm or expanding<br>Interventional or surgical occlusion
